# Selection of appropriate reference genes for RT-qPCR analysis in *Propylea japonica* (Coleoptera: Coccinellidae)

**DOI:** 10.1371/journal.pone.0208027

**Published:** 2018-11-27

**Authors:** Jing Lü, Shimin Chen, Mujuan Guo, Cuiyi Ye, Baoli Qiu, Chunxiao Yang, Huipeng Pan

**Affiliations:** 1 Key Laboratory of Bio-Pesticide Innovation and Application of Guangdong Province/Engineering Technology Research Center of Agricultural Pest Biocontrol in Guangdong Province, South China Agricultural University, Guangzhou, China; 2 State Key Laboratory for Conservation and Utilization of Subtropical Agro-bioresources, South China Agricultural University, Guangzhou, China; Chinese Academy of Agricultural Sciences, CHINA

## Abstract

Reverse transcriptase-quantitative polymerase chain reaction (RT-qPCR) is a reliable technique commonly used in molecular biology to analyze RNA expression. The selection of suitable reference genes for data normalization is a precondition for credible measurements of gene expression levels using RT-qPCR. *Propylea japonica* is one of the most common pests of many crop systems throughout East Asia, and has often been used in the testing of non-target impacts during environmental risk assessments of genetically engineered plants. The present study assessed the suitability of nine frequently used reference genes for comparisons of *P*. *japonica* gene expression. Expression stability was compared across developmental stages, sex, a range of tissues, and following exposure to different temperatures. Data were analyzed using *RefFinder*, which integrated the results obtained using *NormFinder*, *geNorm*, *BestKeeper*, and the *ΔCt* method. This led to the identification of unique sets of reference genes for each experimental condition: *ribosomal protein S18* (*RPS18*) and *elongation factor 1 α* (*EF1A*) for developmental stage comparisons, *RPS18* and *EF1A* for sex comparisons, *EF1A* and *ribosomal protein L4* for tissue comparisons, and *RPS18* and *EF1A* for analyses of temperature-mediated effects. These reference genes will help to enhance the accuracy of RT-qPCR analyses of *P*. *japonica* gene expression. This work represents an initial move towards building a standardized system for RT-qPCR analysis of *P*. *japonica*, providing a basis for the ecological risk assessment of RNAi-based insect control products.

## Introduction

Reverse transcriptase-quantitative PCR (RT-qPCR) is frequently employed as a powerful method for the quantification of gene expression. However, various factors, including RNA quantity and quality, cDNA quantity and quality, and PCR efficiency can significantly influence the quantification cycle (*C*_*q*_) values obtained using this method [1,2]. RT-qPCR data are generally analyzed by normalizing target gene expression to that of one or more suitable reference genes. Although reference gene expression profiles should ideally be stable under the relevant experimental conditions, previous studies have indicated that the expression of many frequently used reference genes can be markedly affected by different treatments [3–11]. Therefore, preliminary evaluations should be conducted to identify stable reference genes for RT-qPCR analysis in a given species under the proposed experimental conditions.

Reference gene selection for RT-qPCR normalization in insect gene expression studies over the past 10 years was recently reviewed for studies that employed the most widely used SYBR Green method [12]. A total of 39 experimental factors were investigated in these papers [12]. Many of these studies employed RNA interference (RNAi), which has been widely used to investigate insect gene function under a range of experimental conditions. RNAi is a biological process in which RNA molecules inhibit protein production by neutralizing targeted mRNA molecules [13]. The mode of action of RNAi-based insecticides and/or RNAi genetically engineered plants suggests that unintended off-target impacts may occur due to altered gene expression in non-target organisms [13,14], and RT-qPCR offers an convenient instrument to detect these gene expression changes. The lady beetle, *Propylaea japonica* (Thunberg) (Coleoptera: Coccinellidae) is one of the most widespread indigenous natural pests in many planting systems in East Asia. As a representative species of predator lady beetles, *P*. *japonica* has been widely used to assess the latent risks of *Bacillus thuringiensis* crops [15–20]. This species is also likely to be selected as a surrogate species to evaluate the effects of RNAi-based insect control products. Because these products may cause lethal or sub-lethal effects on *P*. *japonica* by altering gene expression, it is important to identify suitable reference genes for RT-qPCR analyses of this species.

In this study, we aimed to identify stable reference genes for RT-qPCR analysis in *P*. *japonica*. Nine frequently-used reference genes were investigated: *β-actin* (*Actin*), *glyceraldehyde-3-phosphate dehydrogenase* (*GAPDH*), *α-tubulin* (*TUBA*), *elongation factor 1 α* (*EF1A*), *ribosomal protein L4* (*RPL4*), *ribosomal protein S18* (*RPS18*), *arginine kinase* (*ArgK*), *heat shock protein 90* (*HSP90*), and *vacuolar-type H*^*+*^*-ATPase subunit A* (*V-ATPase A*). All of these reference genes have been used frequently for RT-qPCR analyses in other insects (S1 Table). The stability of each candidate was assessed for four experiments evaluating the effects of developmental stage, sex, tissue, and temperature on gene expression.

## Materials and methods

### Insects

*Propylea japonica* adults were collected in the Mengshan Mountain region (Shandong Province, China) during June 2010 [15]. Since then, the colony has been maintained in the laboratory at a temperature of 26 ± 1°C and a relative humidity of 60–80%, with a 14: 10 h light: dark cycle. They were supplied with *Aphis craccivora* Koch, which were reared on fava bean (*Vicia faba*) in a greenhouse at 20–28°C.

### Experimental conditions

Each *P*. *japonica* developmental stage was sampled on the first day of each stage; this included eggs, four larval instars, pupae, and female and male adults. The numbers of sampled individuals for each replicate in each stage was as follows: 15 eggs; five individuals for the 1^st^ instar; five individuals for the 2^nd^ instar; three individuals for the 3^rd^ instar; one individual for the 4^th^ instar; one pupa; and one female or male individual for adult female or male stages. Different body tissues, including head, midgut, Malpighian tubule, and carcass (body except for the above tissues) were dissected from the 4^th^ instar larvae and female and male adults; about 15 individuals were dissected per replicate. The tissues were stored in RNA*later* (Thermo Fisher Scientific Inc., Waltham, MA, USA) at 4°C until total RNA isolation.

To investigate temperature-mediated effects, three replicate samples of five 1^st^ instars were maintained at 8, 25, or 35°C for 3 h. The samples were then placed in 1.5-ml centrifuge tubes, snap-frozen in liquid nitrogen, and stored at -80°C until total RNA isolation.

### Total RNA extraction and cDNA synthesis

Total RNA samples were extracted from eggs and Malpighian tubules using TRIzol reagent (Invitrogen, Carlsbad, CA, USA), as previously described [20]. Total RNAs were isolated from the other samples using the HiPure Total RNA Micro Kit (Magen, Shanghai, China), in accordance with the manufacturer’s instructions. Gel electrophoresis and the NanoDrop One spectrophotometer (Thermo Fisher Scientific) were used to determine the quantity of total RNA. This was then dissolved in 10–70 μl ddH_2_O to obtain the following RNA concentrations (mean ± standard error of the mean): 200.7 ± 29.4 ng/μl for eggs; 284.4 ± 16.4 ng/μl for first instars; 637.8 ± 52.5 ng/μl for second instars; 563.0 ± 96.8 ng/μl for third instars; 864.3 ± 177.3 ng/μl for fourth instars; 804.9 ± 34.4 ng/μl for pupae; 831.4 ± 88.1 ng/μl for male adults; 866.6 ± 129.6 ng/μl for female adults; 279.8 ± 18.5 ng/μl for heads; 821.2 ± 140.4 ng/μl for carcasses; 359.5 ± 104.1 ng/μl for midguts; 553.2 ± 149.1 ng/μl for Malpighian tubules; 290.67 ± 28.71 ng/μl for first instars at 8°C; 240.57 ± 53.29 ng/μl for first instars at 25°C; and 277.10 ± 53.19 ng/μl for first instars at 35°C. The 260/280 nm optical density ratios were between 1.9 and 2.1 for all samples. The PrimeScript RT kit (containing gDNA Eraser, Perfect Real Time; TaKaRa, Dalian, China) was used to prepare first-strand cDNA for gene expression analysis. The cDNA was diluted tenfold prior to the following RT-qPCR investigations.

### Gene cloning and primer design

A total of nine reference genes were assessed (S1 Table, Table 1). Degenerate primers for *ArgK* had been designed previously [10]. Primers for the other eight genes were designed using the sequences obtained from previous transcriptome datasets [14] (GenBank accession: SRX554957).

**Table 1:** Primers used for RT-qPCR.

Gene	Primer sequences (5’-3’)	Length(bp)	Efficiency (%)	R^2^	Linear regression
*Actin*	F: TGTGCTATGTCGCTTTGG	129	95.4	0.9988	y = -3.4376x+18.887
	R: CTGGGCAACGGAATCTTT				
*GAPDH*	F: GTATCGGTCGTCTTGTACTG	121	93.5	0.9983	y = -3.4879x+22.93
	R: CCATGGGTGGAGTCATATTT				
*EF1A*	F: CTGGAAAGACCACAGAAGAAA	114	91.7	0.9999	y = -3.5381x+18.601
	R: GAGGAGGGAATTCTTGGAAAG				
*TUBA*	F: TGGTTGATAATGAAGCCATCTA	117	99.8	0.9813	y = -3.3267x+23.937
	R: GAGAAGCAGTGATTGAAGAAAC				
*RPL4*	F: CGTCGTCTTAACCCACTTAC	118	91.9	0.9965	y = -3.532x+20.291
	R: CTTCTTCTCTGGCCAACTG				
*RPS18*	F: CGCTGGTGATTCCAGATAAA	111	102.1	0.9992	y = -3.2836x+23.82
	R: GACGACCTACACCTTTGATG				
*HSP90*	F: GTTACCAATCCCTCACCAATC	132	90.2	0.9994	y = -3.5803x+20.565
	R: CTAAATCGGCCTTGGTCATAC				
*ArgK*	F: GACGTTCTTTGGAGGGATAC	102	91.8	0.9999	y = -3.5349x+21.624
	R: CATCGTCGAGTCCAGATAAAG				
*V-ATPase A*	F: CATCTGCCACTCTTGGTATC	120	100.1	0.9987	y = -3.3187x+24.889
	R: CCAAAGCTCTCGTGTACTTC				

PCR reactions were performed using a total volume of 20 μl, as described previously [21]. Amplicons of the expected lengths were purified using the TIANgel Midi Purification Kit (TIANGEN, Beijing, China), and subcloned into the pClone007 Blunt vector before transformation into *Escherichia coli* DH5α competent cells (TSINGKE, Beijing, China) for sequencing by TSINGKE company. Reference gene sequences were confirmed by comparison with the NCBI database.

### RT-qPCR analysis

The RT-qPCR reactions were conducted in accordance with our previous study [21]. The melting curve and standard curve for each candidate gene was also generated as described previously [21]. The RT-qPCR efficiencies (E) were calculated using the following equation: E = (10^[-1/slope]^ - 1) × 100.

### Determination of reference gene expression stability

The stabilities of the nine reference genes were assessed using the following four approaches: *geNorm* [22], *NormFinder* [23], *BestKeeper* [24], and the *ΔCt* method [25]. Finally, the findings of these four analytical tools were integrated by *RefFinder* (http://150.216.56.64/referencegene.php), providing a stability ranking of the candidates. The optimal number of reference genes for target gene normalization was determined by pairwise variation (V_n_/V_n+1_) using V-values calculated by *geNorm* [22]. A V_n_/V_n+1_ cutoff value of ≤ 0.15 signified that the additional n + 1 reference gene was unnecessary; this indicated the appropriate number of reference genes for RT-qPCR data normalization;

## Results

### Candidate gene cloning and performance

**Fig 1:**
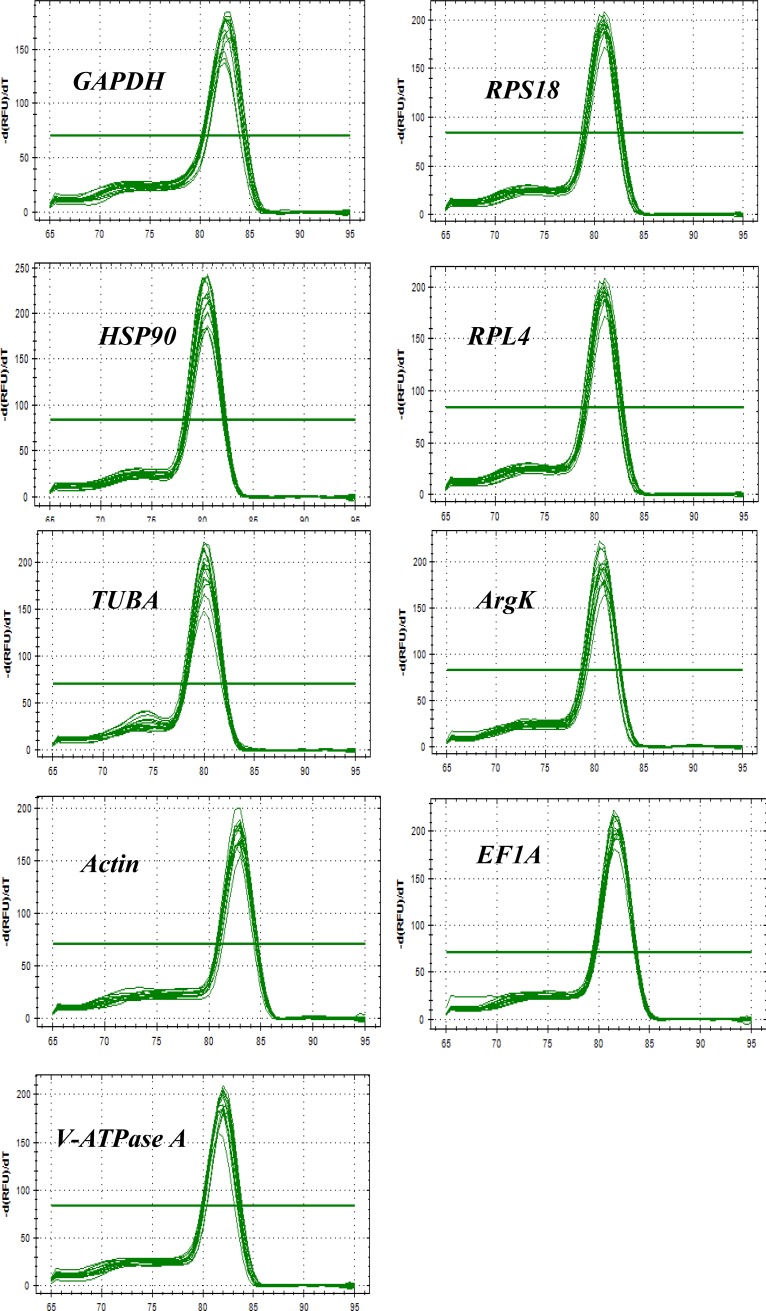
Melting curves of the nine reference genes examined for *Propylea japonica*. *β-actin* (*Actin*), *glyceraldehyde-3-phosphate dehydrogenase* (*GAPDH*), *elongation factor 1 α* (*EF1A*), *α-tubulin* (*TUBA*), *ribosomal protein L4* (*RPL4*), *ribosomal protein S18* (*RPS18*), *heat shock protein 90* (*HSP90*), *arginine kinase* (*ArgK*), and *vacuolar-type H*^*+*^*-ATPase subunit A* (*V-ATPase A*).

All reference genes were expressed in *P*. *japonica* and each was visualized as a single amplicon (S1 Fig). The specific amplification of all reference genes was confirmed by melting curve analyses (Fig 1).

**Fig 2:**
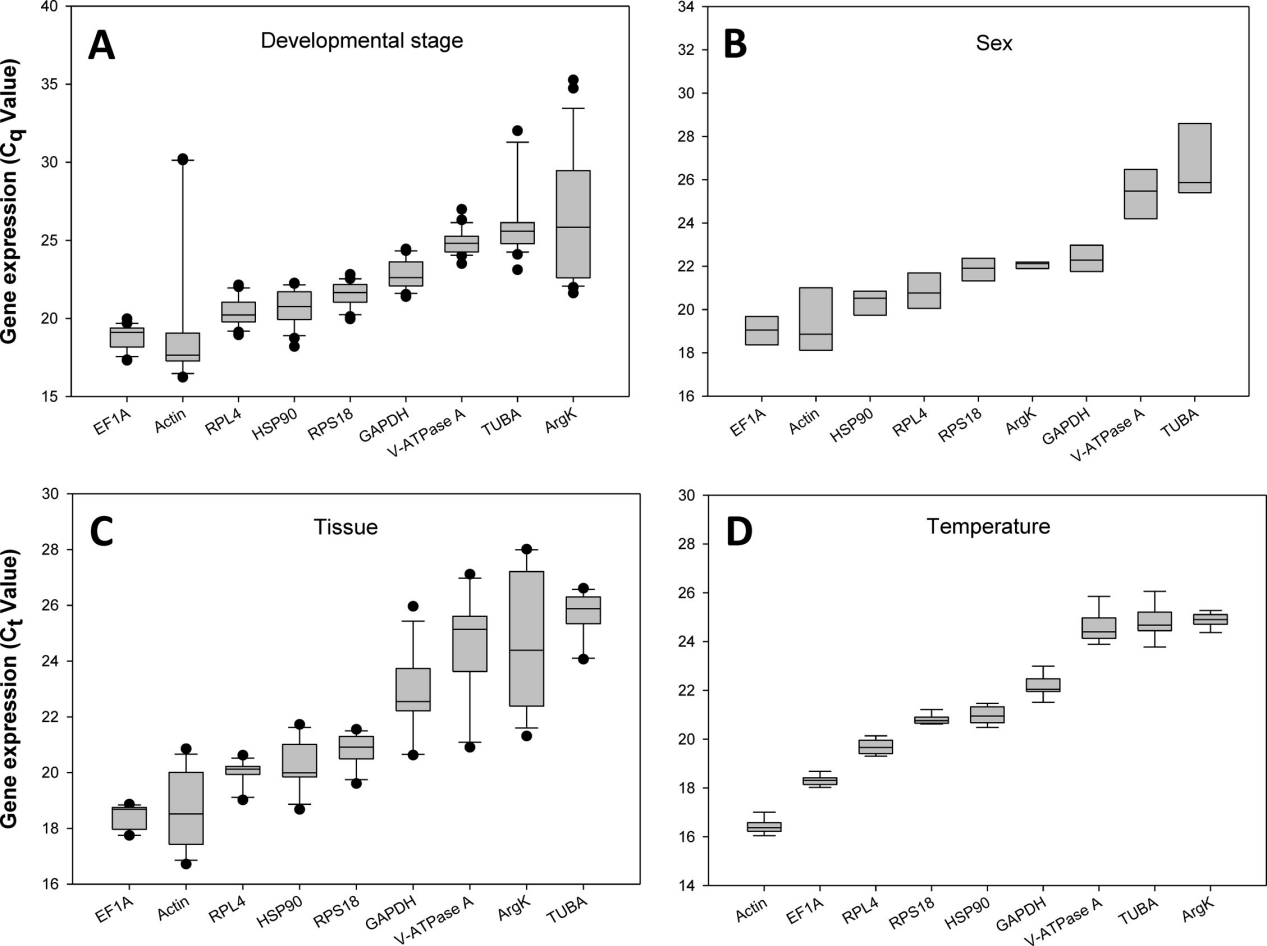
Expression profiles of the nine *Propylea japonica* reference genes. The *Cq* values for each gene are shown for the four experimental conditions. *β-actin* (*Actin*), *glyceraldehyde-3-phosphate dehydrogenase* (*GAPDH*), *elongation factor 1 α* (*EF1A*), *α-tubulin* (*TUBA*), *ribosomal protein L4* (*RPL4*), *ribosomal protein S18* (*RPS18*), *heat shock protein 90* (*HSP90*), *arginine kinase* (*ArgK*), and *vacuolar-type H*^*+*^*-ATPase subunit A* (*V-ATPase A*).

Table 1 shows the E of each PCR, the linear regression equation, and the correlation coefficient (R^2^) for each standard curve. The standard curve for each gene is also shown (S2 Fig). The *C*_*q*_ values for these reference genes under the four experimental situations ranged from 19 to 26. *EF1A* and *Actin* had the highest expression levels, whereas *TUBA* and *ArgK* showed the lowest levels of expression (Fig 2).

### Reference gene expression stability for each experimental condition

Table 2 shows the overall order of gene expression stability determined using *geNorm*, *NormFinder*, *BestKeeper*, and the *ΔC*_*t*_ method, from the most consistent to the least consistent reference genes, under each experimental condition. The major results obtained using each method are indicated below.

**Table 2:** Stability of the expression of nine candidate reference genes in *Propylea japonica* under different experimental conditions calculated by the four different analytical tools *geNorm*, *NormFinder*, *BestKeeper*, and the *ΔCt* method, respectively.

Conditions	CRGs*	*geNorm*		*Normfider*		*BestKeeper*		*ΔCt*	
		Stability	Rank	Stability	Rank	Stability	Rank	Stability	Rank
Developmental	*EF1A*	0.494	2	0.861	4	0.636	3	1.861	1
Stage	*RPS18*	0.366	1	0.641	1	0.582	1	1.871	2
	*Actin*	2.656	8	4.371	9	2.977	8	4.708	9
	*RPL4*	0.366	1	0.907	5	0.730	4	1.936	3
	*ArgK*	2.069	7	3.953	8	3.322	9	4.388	8
	*HSP90*	0.890	5	1.267	6	0.912	6	2.121	6
	*GAPDH*	0.808	4	0.834	3	0.790	5	2.038	5
	*V-ATPase A*	0.713	3	0.719	2	0.599	2	2.005	4
	*TUBA*	1.280	6	2.346	7	1.571	7	2.975	7
Sex	*EF1A*	0.179	1	0.090	1	0.588	5	0.881	1
	*RPS18*	0.260	2	0.123	2	0.487	3	0.887	2
	*Actin*	0.972	7	2.188	7	1.288	8	2.304	8
	*RPL4*	0.350	4	0.185	4	0.705	6	0.971	5
	*ArgK*	0.590	6	0.714	5	0.161	1	1.209	6
	*HSP90*	0.179	1	0.090	1	0.505	4	0.903	3
	*GAPDH*	0.308	3	0.170	3	0.452	2	0.920	4
	*V-ATPase A*	0.468	5	0.870	6	0.983	7	1.232	7
	*TUBA*	1.312	8	2.397	8	1.841	9	2.504	9
Tissue	*EF1A*	0.299	1	0.149	1	0.357	2	1.082	1
	*RPS18*	0.339	2	0.161	2	0.409	3	1.088	2
	*Actin*	1.206	7	1.493	7	1.077	7	1.850	7
	*RPL4*	0.299	1	0.239	3	0.273	1	1.148	3
	*ArgK*	1.557	8	2.637	9	2.199	9	2.786	9
	*HSP90*	0.457	3	0.350	4	0.683	5	1.168	4
	*GAPDH*	0.828	5	1.125	6	1.015	6	1.532	6
	*V-ATPase A*	1.022	6	1.616	8	1.293	8	1.880	8
	*TUBA*	0.670	4	1.024	5	0.580	4	1.477	5
Temperature	*EF1A*	0.082	1	0.031	2	0.149	2	0.326	1
	*RPS18*	0.082	1	0.124	4	0.137	1	0.343	3
	*Actin*	0.152	2	0.051	3	0.201	4	0.342	2
	*RPL4*	0.179	3	0.028	1	0.247	5	0.350	4
	*ArgK*	0.228	4	0.305	5	0.200	3	0.431	5
	*HSP90*	0.275	5	0.308	6	0.298	6	0.453	6
	*GAPDH*	0.316	6	0.412	7	0.323	7	0.506	7
	*V-ATPase A*	0.381	7	0.550	8	0.460	8	0.617	8
	*TUBA*	0.452	8	0.649	9	0.464	9	0.700	9

* Candidate reference gene

#### geNorm

Across different developmental stages, *RPS18* and *RPL4* were both ranked as the most stable genes, while *EF1A* and *HSP90* were ranked together as the most stable genes in the sex comparison. For the tissue comparisons, *EF1A* and *RPL4* were both ranked as the most stable genes, while *EF1A* and *RPS18* were ranked together as the most stable genes in the temperature experiment.

#### NormFinder

Across different developmental stages, *RPS18* was the most stable gene. In females and males, *EF1A* and *HSP90* were ranked together as the most stable genes. Among different tissues, *EF1A* ranked as the most stable gene, while *RPL4* showed the most stable expression in the temperature experiment.

#### BestKeeper

Across different developmental stages, *RPS18* was the most stable gene, while *ArgK* showed the most stable expression in females and males. For the tissue comparisons, *RPL4* was the most stable gene, whereas *RPS18* showed the most stable expression at different temperatures.

#### The ΔC_t_ method

The average standard deviation of each gene set was negative correlated with its stability. The most stable gene under each of the four experimental conditions was *EF1A*.

### The overall *RefFinder* ranking of reference gene expression stability

**Fig 3:**
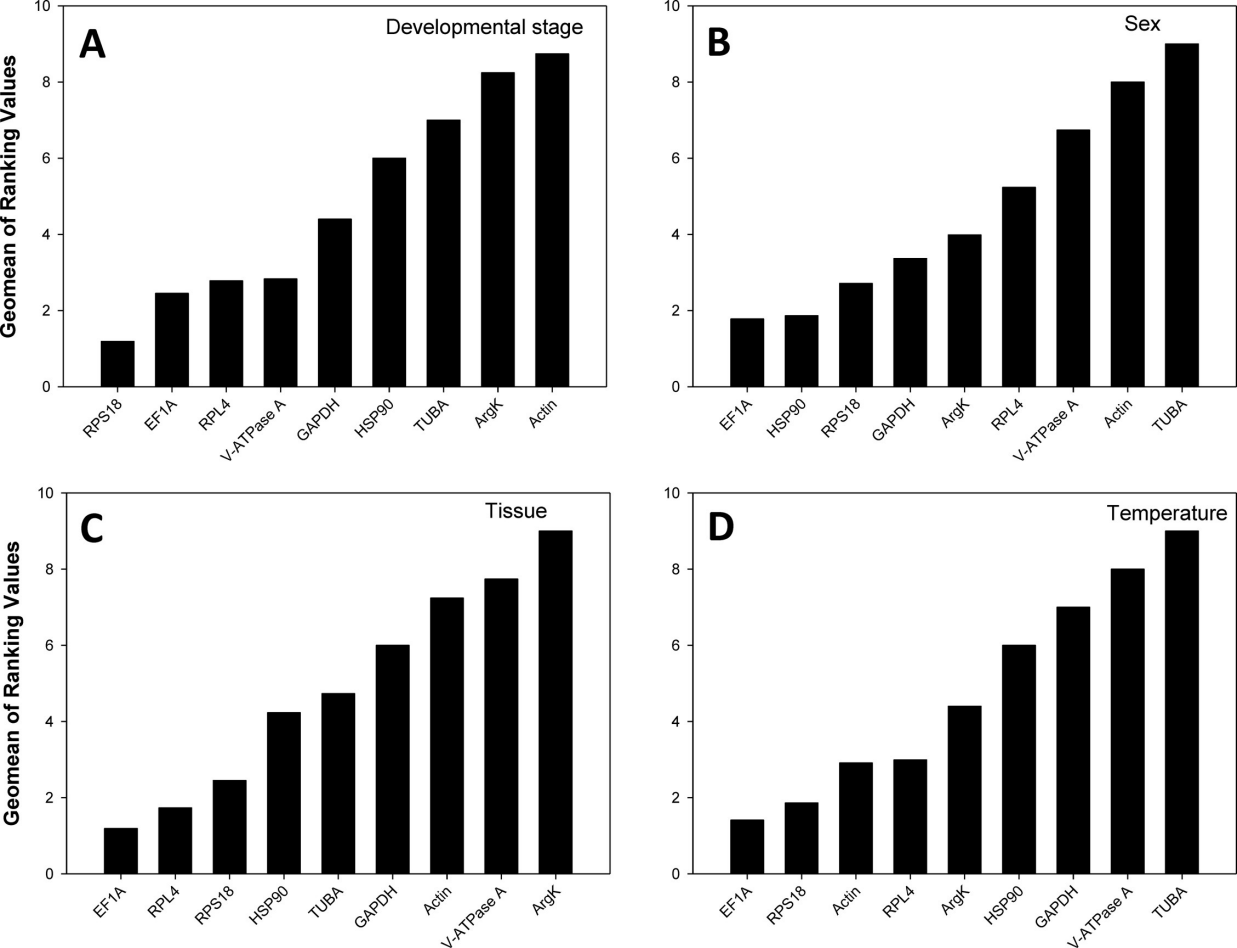
Stability of the nine *Propylea japonica* reference genes according to *RefFinder*. A lower *Geomean* value indicates a more stable expression. *β-actin* (*Actin*), *glyceraldehyde-3-phosphate dehydrogenase* (*GAPDH*), *elongation factor 1 α* (*EF1A*), *α-tubulin* (*TUBA*), *ribosomal protein L4* (*RPL4*), *ribosomal protein S18* (*RPS18*), *heat shock protein 90* (*HSP90*), *arginine kinase* (*ArgK*), and *vacuolar-type H*^*+*^*-ATPase subunit A* (*V-ATPase A*).

The comprehensive reference gene rankings for expression stability under each experimental condition are shown in Fig 3.

### Optimal number of reference genes for target gene normalization based on *geNorm*

**Fig 4:**
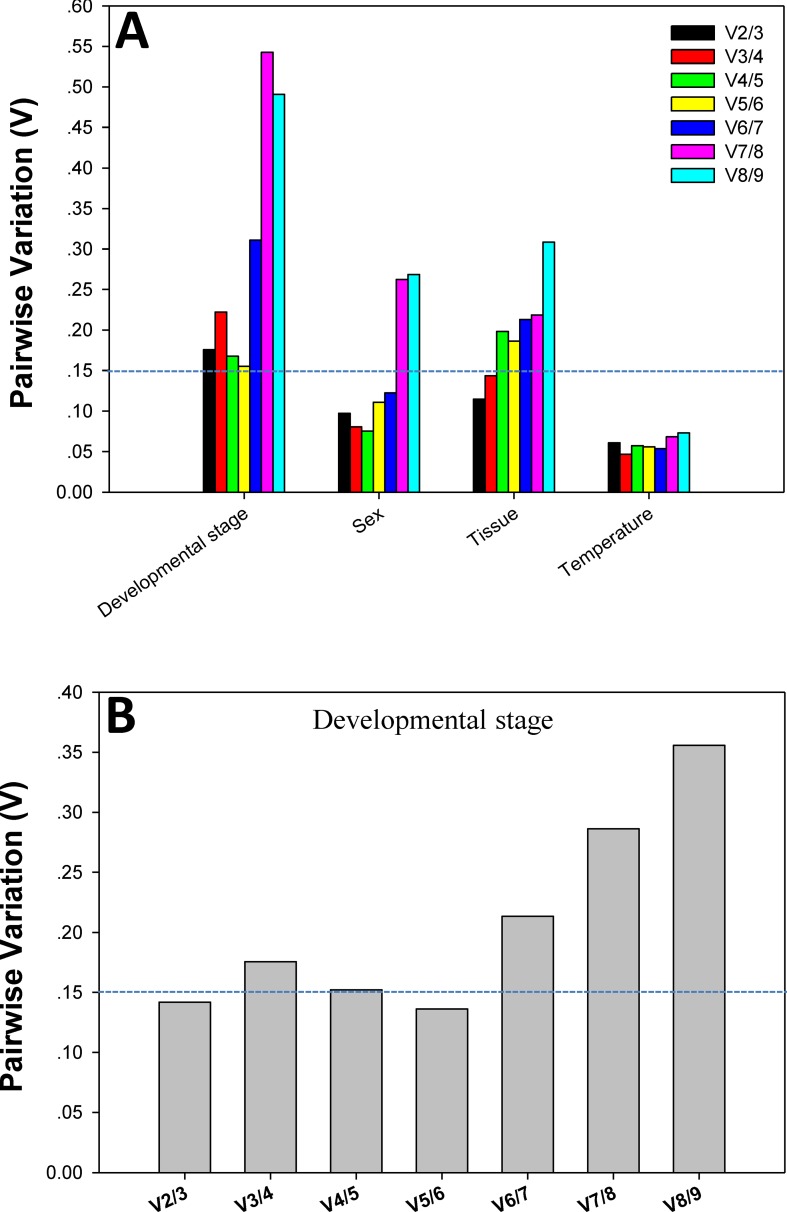
Pairwise variation (V) values determined using *geNorm*. (**A**) Across the four indicated comparisons, and (**B**) for the comparison across developmental stage only.

Although V-values for the analyses of developmental stage were never < 0.15, V2/3 was lower than V3/4 (Fig 4A). In addition, the expression of *Actin* and *ArgK* was obviously lower at the egg stage than at other stages. When the egg stage data were removed, and data from the remaining stages were analyzed, we found that V2/3 was < 0.15. Therefore, the two most stable candidates, *RPS18* and *EF1A*, were recommended for data normalization across different developmental stages (Fig 4B). For the sex, tissue, and temperature comparisons, the first V-values < 0.15 emerged at V2/3, indicating that two reference genes were adequate for normalization under these experimental conditions. Thus, the nominated reference genes were *EF1A* and *HSP90* for sex, *EF1A* and *RPL4* for tissue comparisons, and *EF1A* and *RPS18* for analyses of temperature-mediated effects on gene expression.

## Discussion

Taken together with previous studies, the present findings clearly showed that reference gene expression stability is condition-specific and, accordingly, no one gene can be used to normalize all RT-qPCR data. This strongly indicates the need to conduct customized reference gene selection for RT-qPCR analyses under the relevant experimental conditions, even for the same species. For example, six papers relating to reference gene selection for the whitefly, *Bemisia tabaci*, have been published over the past five years [5, 26–30].

The key recommendations for reference gene selection in the MIQE guidelines state that at least two reference genes should be employed in order to avoid biased normalization [31]. The optimal number of reference genes is typically determined by *geNorm*; based on these results, two reference genes were adequate for the experimental conditions employed in the present study. These results were partly in accordance with those reported previously for other lady beetle species [9–11, 32].

Previous investigations have demonstrated that there is no single reference gene that is applicable under all experimental conditions [1, 2, 5–11]. For example, *Actin* is a major structural protein that is often used as an internal control without prior validation. However, the present study found that *Actin* was one of the least stable reference genes under the test conditions; this is consistent with previous studies of four other Coccinellidae species [9–11, 32]. Therefore, we suggest that researchers should initially select reference genes that have been verified within the same family or genus as their target species.

As far as we know, the present study is the first to identify stable RT-qPCR reference genes in *P*. *japonica*. Sets of two reference genes were nominated for each experimental condition: *EF1A* and *RPS18* for comparisons between different developmental stages; *EF1A* and *HSP90* for comparisons of female and male adults; *EF1A* and *RPL4* for comparisons of different tissues; and *EF1A* and *RPS18* for investigation of temperature-mediated effects. This study represents an initial move towards building a standardized system for RT-qPCR analyses in *P*. *japonica*. This will inform ecological risk assessments of RNAi-based insect control products on *P*. *japonica*, and facilitate in-depth functional genomic studies of *P*. *japonica*.

## Supporting information

S1 Fig Agarose gel electrophoresis of the nine candidate reference genes. PCR reaction templates: 1) *β-actin* (*Actin*); 2) *glyceraldehyde-3-phosphate dehydrogenase* (*GAPDH*); 3) *elongation factor 1 α* (*EF1A*); 4) *α-tubulin* (*TUBA*); 5) *ribosomal protein L4* (*RPL4*); 6) *ribosomal protein S18* (*RPS18*); 7) *heat shock protein 90* (*HSP90*); 8) *arginine kinase* (*ArgK*); 9) *vacuolar-type H*^*+*^*-ATPase subunit A* (*V-ATPase A*). M, DL100 DNA marker.
(TIFF)

S2 Fig Standard curves of the nine candidate reference genes. (TIFF)

S1 Table Nucleotide sequences of the nine reference genes. (DOC)

## References

[1] Bustin SA, Benes V, Nolan T, Pfaffl MW (2005) Quantitative real-time RT-PCR-a perspective. J Mol Endocrinol 34: 597–601. 10.1677/jme.1.01755 15956331 10.1677/jme.1.01755

[2] Strube C, Buschbaum S, Wolken S, Schnieder T (2008) Evaluation of reference genes for quantitative real-time PCR to investigate protein disulfide isomerase transcription pattern in the bovine lungworm *Dictyocaulus viviparus*. Gene 425: 36–43. 10.1016/j.gene.2008.08.001 18761062 10.1016/j.gene.2008.08.001

[3] Chapman JR, Waldenström J (2015) With reference to reference genes: a systematic review of endogenous controls in gene expression studies. PLoS One 10: e0141853 10.1371/journal.pone.0141853 26555275 10.1371/journal.pone.0141853 PMC4640531

[4] Hellemans J, Vandesompele J (2014) Selection of reliable reference genes for RT-qPCR analysis In Quantitative Real-Time PCR (pp. 19–26). Springer New York. 10.1007/978-1-4939-0733-5_3 24740218

[5] Li RM, Xie W, Wang SL, Wu QJ, Yang NN, Yang X, et al (2013) Reference gene selection for qRT-PCR analysis in the sweetpotato whitefly, *Bemisia tabaci* (Hemiptera: Aleyrodidae). PLoS One 8: e53006 10.1371/journal.pone.0053006 23308130 10.1371/journal.pone.0053006 PMC3540095

[6] Yang CX, Pan HP, Liu Y, Zhou XG (2014) Selection of reference genes for expression analysis using quantitative real-time PCR in pea aphid, *Acyrthosiphon pisum* (Harris) (Hemiptera, Aphidiae). PLoS One 9: e110454 10.1371/journal.pone.0110454 25423476 10.1371/journal.pone.0110454 PMC4244036

[7] Yang CX, Pan HP, Liu Y, Zhou XG (2015) Stably expressed housekeeping genes across developmental stages in the two-spotted spider mite, *Tetranychus urticae*. PLoS One 10: e0120833 10.1371/journal.pone.0120833 25822495 10.1371/journal.pone.0120833 PMC4379063

[8] Yang CX, Pan HP, Liu Y, Zhou XG (2015) Temperature and development impacts on housekeeping gene expression in cowpea aphid, *Aphis craccivora* (Hemiptera: Aphidiae). PLoS One 10: e0130593 10.1371/journal.pone.0130593 26090683 10.1371/journal.pone.0130593 PMC4474611

[9] Yang CX, Pan HP, Noland JE, Zhang DY, Zhang ZH, Liu Y, et al (2015) Selection of reference genes for RT-qPCR analysis in a predatory biological control agent, *Coleomegilla maculata* (Coleoptera: Coccinellidae). Sci Rep 5: 18201 10.1038/srep18201 26656102 10.1038/srep18201 PMC4674751

[10] Yang CX, Preisser EL, Zhang HJ, Liu Y, Dai LY, Pan HP, et al (2016) Selection of reference genes for RT-qPCR analysis in *Coccinella septempunctata* to assess un-intended effects of RNAi transgenic plants. Front Plant Sci 7: 1672 10.3389/fpls.2016.01672 27877186 10.3389/fpls.2016.01672 PMC5099537

[11] Yang XW, Pan HP, Yuan L, Zhou X (2018) Reference gene selection for RT-qPCR analysis in *Harmonia axyridis*, a global invasive lady beetle. Sci Rep 8: 2689 10.1038/s41598-018-20612-w 29426915 10.1038/s41598-018-20612-w PMC5807316

[12] Lü J, Yang CX, Zhang YJ. Pan, HP (2018) Selection of reference genes for the normalization of RT-qPCR data in gene expression studies in insects: a systematic review. Front Physiol 9: 1560. 30459641 10.3389/fphys.2018.01560 PMC6232608

[13] Berezikov E (2011) Evolution of microRNA diversity and regulation in animals. Nat Rev Genet 12: 846–860. 10.1038/nrg3079 22094948 10.1038/nrg3079

[14] Baum JA, Bogaert T, Clinton W, Heck GR, Feldmann P, Ilagan O, et al (2007) Control of coleopteran insect pests through RNA interference. Nat Biotechnol 25: 1322–1326. 10.1038/nbt1359 17982443 10.1038/nbt1359

[15] Tang LD, Wang XM, Jin FL, Qiu BL, Wu JH, Ren SX (2014) *De novo* sequencing-based transcriptome and digital gene expression analysis reveals insecticide resistance-relevant genes in *Propylaea japonica* (Thunberg) (Coleoptea: Coccinellidae). PLoS One 9: e100946 10.1371/journal.pone.0100946 24959827 10.1371/journal.pone.0100946 PMC4069172

[16] Li Y, Liu Y, Yin X, Romeis J, Song X, Chen X, et al (2017) Consumption of Bt maize pollen containing Cry1Ie does not negatively affect Propylea japonica (Thunberg) (Coleoptera: Coccinellidae). Toxins 9: 108. 10.3390/toxins9030108 PMC5371863 28300767

[17] Li Y, Zhang X, Chen X, Romeis J, Yin X, Peng Y (2015) Consumption of Bt rice pollen containing Cry1C or Cry2A does not pose a risk to *Propylea japonica* (Thunberg) (Coleoptera: Coccinellidae). Sci Rep 5: 7679 10.1038/srep07679 25567127 10.1038/srep07679 PMC4286735

[18] Li Y, Liu Q, Wang Y, Chen X, Song X, Romeis J, et al (2016) Ingestion of Bt corn pollen containing Cry1Ab/2Aj or Cry1Ac does not harm *Propylea japonica* larvae. Sci Rep 6: 23507 10.1038/srep23507 27005950 10.1038/srep23507 PMC4804303

[19] Zhang X, Li Y, Romeis J, Yin X, Wu K, Peng Y (2014) Use of a pollen-based diet to expose the ladybird beetle *Propylea japonica* to insecticidal proteins. PLoS One 9: e85395 10.1371/journal.pone.0085395 24409328 10.1371/journal.pone.0085395 PMC3883695

[20] Zhao Y, Zhang S, Luo JY, Wang CY, Lv LM, Wang XP, et al (2016) Bt proteins Cry1Ah and Cry2Ab do not affect cotton aphid *Aphis gossypii* and ladybeetle *Propylea japonica*. Sci Rep 6: 20368 10.1038/srep20368 26829252 10.1038/srep20368 PMC4734323

[21] Pan H, Yang X, Bidne K, Hellmich RL, Siegfried BD, Zhou X (2015) Selection of reference genes for RT-qPCR analysis in the monarch butterfly, *Danaus plexippus* (L.), a migrating bio-indicator. PLoS One 10: e0129482 10.1371/journal.pone.0129482 26030778 10.1371/journal.pone.0129482 PMC4452232

[22] Vandesompele J, De Preter K, Pattyn F, Poppe B, Van Roy N, De Paepe A, et al (2002) Accurate normalization of real-time quantitative RT-PCR data by geometric averaging of multiple internal control genes. Genome Biol 3: research0034 12184808 10.1186/gb-2002-3-7-research0034 PMC126239

[23] Andersen CL, Jensen JL, Ørntoft TF (2004) Normalization of real-time quantitative reverse transcription-PCR data: a model-based variance estimation approach to identify genes suited for normalization, applied to bladder and colon cancer data sets. Cancer Res 64: 5245–5250. 10.1158/0008-5472.CAN-04-0496 15289330 10.1158/0008-5472.CAN-04-0496

[24] Pfaffl MW, Tichopad A, Prgomet C, Neuvians TP (2004) Determination of stable housekeeping genes, differentially regulated target genes and sample integrity: BestKeeper–Excel-based tool using pair-wise correlations. Biotechnol Lett 26: 509–515. 15127793 10.1023/b:bile.0000019559.84305.47

[25] Silver N, Best S, Jiang J, Thein SL (2006) Selection of housekeeping genes for gene expression studies in human reticulocytes using real-time PCR. BMC Mol Biol 7: 33 10.1186/1471-2199-7-33 17026756 10.1186/1471-2199-7-33 PMC1609175

[26] Collins C, Patel MV, Colvin J, Bailey D, Seal S, Wolfner M (2014) Identification and evaluation of suitable reference genes for gene expression studies in the whitefly *Bemisia tabaci* (Asia I) by reverse transcription quantitative real time PCR. J Insect Sci 14: 63 10.1093/jis/14.1.63 25373210 10.1093/jis/14.1.63 PMC4207516

[27] Dai TM, LüZC, Liu WX, Wan FH (2017) Selection and validation of reference genes for qRT-PCR analysis during biological invasions: The thermal adaptability of *Bemisia tabaci* MED. PLoS One 12: e0173821 10.1371/journal.pone.0173821 28323834 10.1371/journal.pone.0173821 PMC5360248

[28] Liang P, Guo Y, Zhou X, Gao X (2014) Expression profiling in *Bemisia tabaci* under insecticide treatment: indicating the necessity for custom reference gene selection. PLoS One 9: e87514 10.1371/journal.pone.0087514 24498122 10.1371/journal.pone.0087514 PMC3909111

[29] Lü ZH, Pan HP, Zhang W, Ding TB, Chu D (2018) Reference gene selection for RT-qPCR analysis in two invasive whiteflies after the acquisition of vectored or non-vectored viruses. J Asia-Pac Entomol 21: 19–24.

[30] Su YL, He WB, Wang J, Li JM, Liu SS, Wang XW (2013) Selection of endogenous reference genes for gene expression analysis in the Mediterranean species of the *Bemisia tabaci* (Hemiptera: Aleyrodidae) complex. J Econ Entomol 106: 1446–1455 (2013). 10.1603/EC12459 23865213 10.1603/EC12459

[31] Bustin SA, Benes V, Garson JA, Hellemans J, Huggett J, Kubista M, et al (2009) The MIQE guidelines: minimum information for publication of quantitative real-time PCR experiments. Clin Chem 55: 611–622. 10.1373/clinchem.2008.112797 19246619 10.1373/clinchem.2008.112797

[32] Pan HP, Yang XW, Siegfried BD, Zhou XG (2015) A comprehensive selection of reference genes for RT-qPCR analysis in a predatory lady beetle, *Hippodamia convergens* (Coleoptera: Coccinellidae). PLoS One 10: e0125868 10.1371/journal.pone.0125868 25915640 10.1371/journal.pone.0125868 PMC4411045

