# Supplementary figures and images for: Selection of appropriate reference genes for RT-qPCR analysis in Propylea japonica (Coleoptera: Coccinellidae)
Source: PLoS One. 2018 Nov 27;13(11):e0208027. doi: 10.1371/journal.pone.0208027 (PMC6258549; doi:10.1371/journal.pone.0208027)

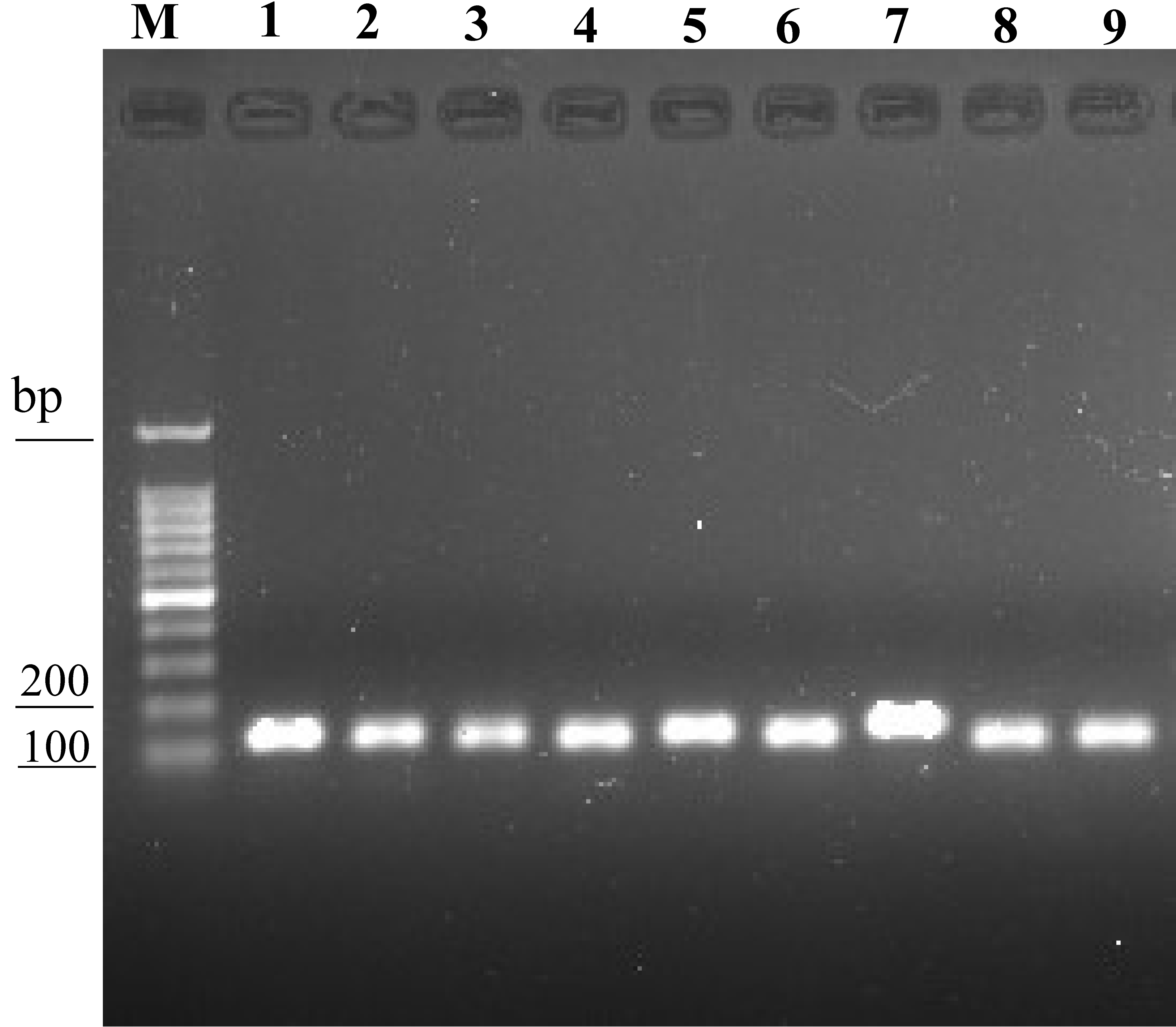

Supplement: S1 Fig — PCR reaction templates: 1) β-actin (Actin); 2) glyceraldehyde-3-phosphate dehydrogenase (GAPDH); 3) elongation factor 1 α (EF1A); 4) α-tubulin (TUBA); 5) ribosomal protein L4 (RPL4); 6) ribosomal protein S18 (RPS18); 7) heat shock protein 90 (HSP90); 8) arginine kinase (ArgK); 9) vacuolar-type H+-ATPase subunit A (V-ATPase A). M, DL100 DNA marker. (TIFF) [file pone.0208027.s001.tiff]

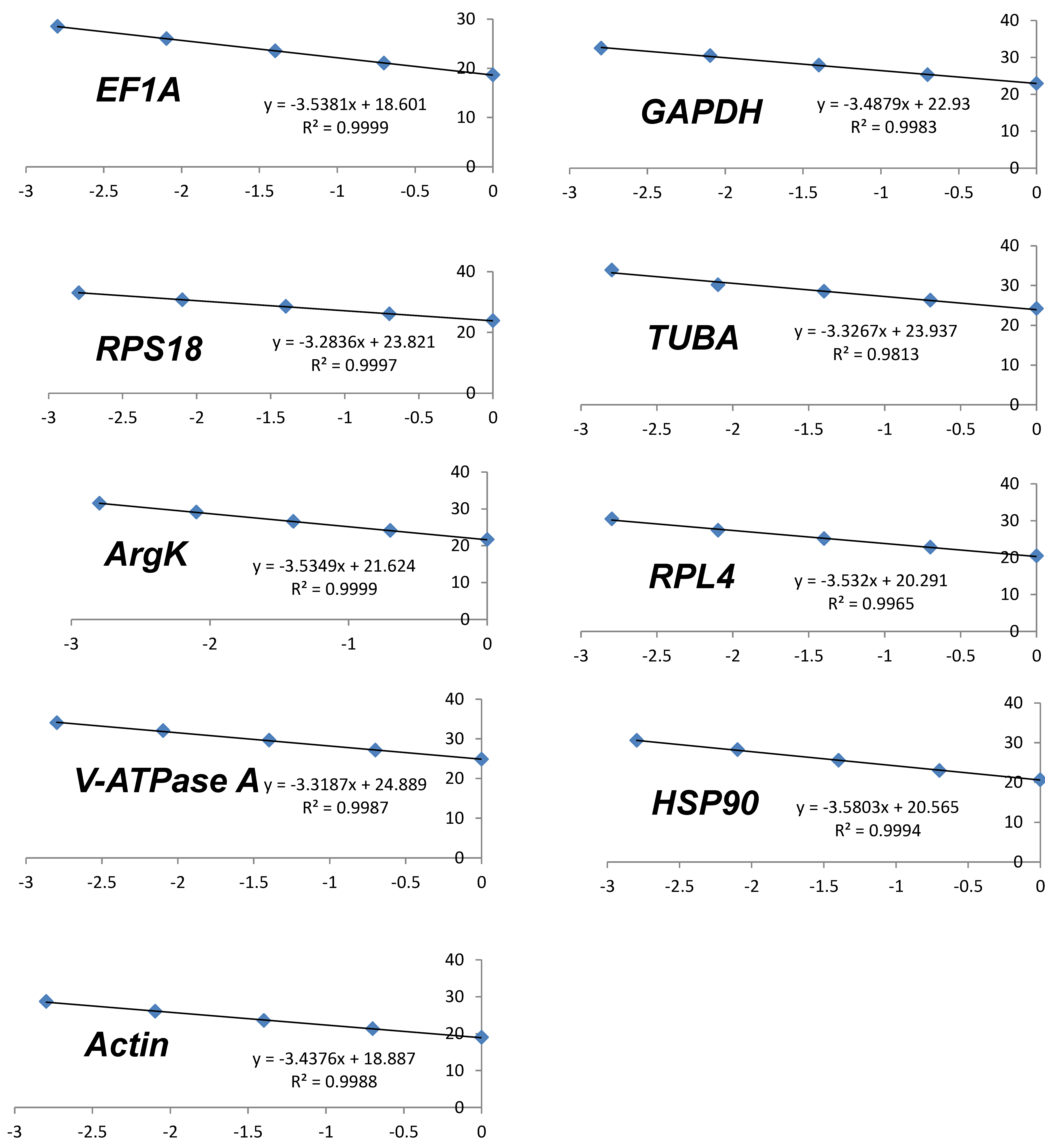

Supplement: S2 Fig — (TIFF) [file pone.0208027.s002.tiff]
