# Supplementary material for: Selection of appropriate reference genes for RT-qPCR analysis in Propylea japonica (Coleoptera: Coccinellidae)
Source: PLoS One. 2018 Nov 27;13(11):e0208027. doi: 10.1371/journal.pone.0208027 (PMC6258549; doi:10.1371/journal.pone.0208027)
Supplement: S1 Table — (DOC) [file pone.0208027.s003.doc]

**Table S1. The nucleotide sequences of these 10 reference genes.**

| Genes | Nucleotide sequences |
| --- | --- |
| *Actin* | GCTCCAGAAGAACACCCTGTACTTCTTACCGAAGCTCCATTGAACCCTAAGGCTAACAGGGAAAAGATGACACAGATTATGTTTGAAACCTTCAACACCCCTGCTATGTACGTTGCAATTCAAGCCGTACTTTCCTTGTACGCTTCTGGTCGTACCACTGGTATCGTCTTGGACTCTGGTGATGGTGTATCTCACACCGTACCAATCTACGAAGGTTACGCACTTCCCCATGCCATCCTCCGTCTTGACTTGGCTGGTCGTGACTTGACCGACTACCTCATGAAGATCCTCACCGAAAGGGGTTACTCTTTCACCACCACAGCTGAAAGGGAAATCGTCCGTGACATCAAGGAAAAATTGTGCTATGTCGCTTTGGACTTCGAACAAGAAATGGCCACCGCTGCTGCCTCCACCTCCTTGGAAAAATCCTATGAACTTCCTGATGGTCAAGTAATCACCATTGGTAACGAAAGATTCCGTTGCCCAGAAGCCCTCTTCCAACCTTCCTTCTTGGGTATGGAATCCTGCGGTATCCACGAAACCGTATACAACTCCATCATGAAGTGCGACGTTGATATCCGTAAGGACTTGTACGCCAACACCGTCTTGTCTGGTGGTACCACCATGTACCCAGGTATCGCTGATCGTATGCAAAAGGAAATCACCGCCTTGGCCCCATCCACCATCAAGATCAAGATCATCGCTCCCCCAGAAAGGAAATACTCCGTCTGGATCGGAGGATCCATCTTGGCTTCCTTGTCCACCTTCCAACAGATGTGGATCTCCAAACAAGAATA |
| *GAPDH* | CTAAATTTGGTATCAACGGTTTCGGACGTATCGGTCGTCTTGTACTGAGGGCTTCCTTGGAAAGGGGAGCAGAATGCGTTGGTGTCAATGACCCTTTCCTCGATGCTGCCTATATGGAATATCTCTTCAAATATGACTCCACCCATGGACGCTTCAAGGGCGAAGTCAGCCATGATGACAAGAACCTAATCATCAACGGTAAAAAGATTGCTATCTTCAGCGAAAGAGACCCCAAAGCCATCCCATGGGGCAAAGTTGGTGCCAAATACGTCGTAGAATCAACTGGAGTCTTCACCACCACCGAGAAGGCCAAGGCTCACATAGAGGGAGGTGCTGTTAAGGTTATCATCTCAGCACCATCTGCCGATGCCCCCATGTATGTTTGCGGTGTCAACCTTGATGAATACGATCCTAAAGCTCCAGTCATCTCAAATGCTTCCTGTACAACCAACTGCTTGGCCCCATTGGCCAAGGTCATCCATGACAAGTTCGAAATTGTTGAAGGTCTTATGACCACCGTACACGCAACCACTGCCACCCAGAAGACCGTTGACGGTCCTTCAGGAAAATTGTGGCGTGATGGTCGTGGTGCTGCCCAGAACATCATTCCAGCCTCCACCGGAGCTGCCAAGGCTGTGACTAAAGTCATCAAGTCTTTGGATGGAAAGTTGACCGGTATGGCTTTCCGTGTCCCAACTGCTGATGTCTCCGTTGTAGACTTGACCTGCCGTCTTGGCAAGGGAGCTTCCTATGACGAAATCAAAAAGGCAGTAAAAGAAGCCGCTGAAGGCCCAATGAAGGGAATTTTGGGATACACAGAAGACGATGTTGTATCATCAGATTTCATTGGAGACTCTCACTCATCGATCTTCGATGCTAAAGCCGGAATTTCTCTAAACCCCAACTTTGTGAAACTCATTGCGTGGTACGACAATGAATACGGATACTCCTGCAGAGTTGTCGACCTCATCAAACACATCAGCACAAAGGA |
| *EF1A* | AATGGGTAAAGAAAAGACTCATATTAACATCGTCGTCATTGGACACGTAGATTCTGGTAAATCTACCACCACAGGTCACTTGATCTACAAATGCGGTGGTATCGACAAACGTACCATCGAAAAATTCGAAAAGGAAGCCCAGGAAATGGGAAAAGGTTCCTTCAAATATGCGTGGGTACTTGACAAACTTAAGGCTGAACGTGAACGTGGTATCACCATCGATATCGCTTTATGGAAATTCGAAACCTCCAAATACTACGTAACCATCATCGATGCCCCTGGACACAGAGATTTCATCAAAAACATGATCACCGGTACATCTCAAGCCGATTGTGCTGTCTTGATCGTAGCTGCTGGTACTGGTGAATTCGAAGCCGGTATCTCAAAGAACGGACAAACTCGTGAGCACGCTCTGCTCGCCTTCACCTTGGGTGTGAAACAACTCATTGTTGGTGTCAACAAAATGGACTCCACCGAACCACCATACAGTGAATCTCGTTTCGAGGAAATCAAAAAGGAAGTATCTTCATACATCAAGAAGATCGGTTACAACCCAGCAGCTGTTGCTTTCGTACCAATCTCTGGATGGCACGGAGACAACATGTTGGAAGCATCCACAAAAATGCCATGGTTCAAGGGATGGGCCATTGAACGTAAAGAAGGAAAGGCTGACGGTAAATGTTTGATCGAAGCTTTGGATGCCATCCTCCCTCCATCTCGTCCAACTGAAAAACCCCTCCGTCTTCCACTCCAGGACGTCTACAAAATTGGAGGTATTGGAACAGTACCAGTAGGTCGTGTTGAAACTGGTGTATTGAAACCAGGTACCGTAGTTGTCTTTGCCCCAGCCAACATTACCACTGAAGTAAAATCCGTTGAAATGCACCACGAAGCCCTTGTGGAAGCTGTACCCGGTGACAACGTTGGTTTCAACGTTAAGAACGTATCAGTGAAAGAATTGCGTCGTGGATACGTCGCTGGAGACTCCAAGAACAACCCACCCAGGGGAGCTTCAGACTTCACTGCCCAAGTAATTGTATTGAACCACCCCGGTCAAATTAGCAACGGATACACCCCAGTTTTGGATTGTCACACAGCCCACATTGCCTGCAAATTCGCCGAAATTAAAGAAAAAGTCGACCGTCGTTCTGGAAAGACCACAGAAGAAAACCCCAAAGCCATCAAATCTGGAGATGCAGCCATCGTCAATTTGGTACCATCCAAGCCAATGTGCGTAGAATCTTTCCAAGAATTCCCTCCTCTAGGTCGTTTCGCTGTCCGTGACATGAGGCAAACCGTTGCTGTAGGAGTTATCAAGAGTGTTAGTTTCAAGGAAGCCGGAGCCGGTAAAGTTACGAAAGCCGCTGAAAAAGCAACGAAGAAGAAGTA |
| *TUBA* | CTCACACAACTCTTGAACACAGCGACTGTGCATTCATGGTTGATAATGAAGCCATCTATGACATTTGCCGTCGTAATCTTGATATTGAACGTCCAACTTACACTAACTTGAATCGTCTCATTGGCCAGATTGTTTCTTCAATCACTGCTTCTCTCCGTTTTGATGGTGCCCTTAATGTTGATCTGACTGAATTCCAGACCAATTTGGTTCCATACCCCCGTATCCATTTCCCGTTGGTCACCTATGCCCCAGTCATCTCTGCTGAAAAGGCTTACCATGAACAATTGTCCGTATCAGAAATCACCAACGCTTGTTTTGAACCAGCCAACCAAATGGTGAAATGTGACCCACGTCATGGCAAATACATGGCTTGTTGTATGTTGTACCGTGGTGATGTCGTCCCCAAGGATG |
| *TUBB* | TCAACACTATAAGGGCGATTGGGCCCTCTAGATGCATGCTCGAGCGGCCGCCAGTGTGATGGATATCTGCAGAATTGCCCTTAACTGGAACGAATCAACGTGTACTTCAATGAGGCAACAGGCGGCAAATACGTACCCAGGGCTGTCTTGGTCGACTTGGAACCTGGCACCATGGATTCCGTGCGCTCAGGACCCTTCGGTCAAATATTTCGACCAGACAACTTCGTATTCGGTCAAAGCGGAGCCGGAAACAACTGGGCCAAAGGACATTACACCGAAGGAGCTGAGCTGGTTGACTCAGTCCTAGACGTCGTTCGTAAAGAAGCAGAAAGCTGCGATTGCATGCAGGGCTTTCAACCGACCCATTCTCTCGGAGGAGGTACAGGTTCTGGCCTTGGTACTCTGTTGATCTCCAAGATTCGCGAGGAGTACCCTGATAGGATTATGAACACGTTTTCTGTTGTACCTTCGCCTAAAGTTTCAGATACTGTGGTAGAGCCTTATAACGCCACTCTTTCCGTCCATCAGCTGGTGGAAAACACTGACGAGACGTACTGCATCGATAACGAGGCCTTGTATGACATCTGCTTCAGGACTTTGAAGCTCACGACCCCTACCTACGGCGACTTGAACCATTTGGTTTCCGCTACCATGTCTGGAGTGACCACTTGCCTACGATTTCCAGGACAACTTAATTCTGATTTGCGTAAATTGGCCGTGAATATGGTTCCTTTTCCGCGACTGCACTTCTTCATGCCGGGTTTTGCGCCTTTAACCTCCCGCGGTAGTCAACAGTATAGGGCTCTAACGGTTCCAGAGCTGGTACTGCAAATGTTTGACGCCAAGAACATGATGGCTGCTTGCGACCCTAGACACGGAAGATACCTAACCGTAGCCGGCATTTTCAAAGGCAAATGGCCA |
| *RPL4* | CCAGCTGTATTTAAGGCCCCAATCAGGCCTGATGTTGTCTCTTTCGTACAACAGCAGGTATCGATGAACCACAGACAGCCCTATTGTGTTAGCGAGAAAGCTGGTCACCAAACATCTGCCGAATCATGGGGTACCGGTAGGGCTGTTGCCCGTATCCCTCGTGTCCGAGGTGGTGGTACTCACCGTTCGGGTCAGGGTGCTTTCGGTAACATGTGTAGAGGAGGTCGCATGTTCGCCCCCACCAAACCATGGCGTAGGTGGCACCGTAGGGTCAACATCAACCAAAGGAGGTACGCTTTGGCCTCTGCCATCGCAGCCAGTGGCGTCCCAGCCCTGGTTATGAGCAAAGGACACGTCATCGAAAATGTTCCAGAATTCCCTCTGGTCGTTTCAGACAAAGTGCAGGAATTGTCCAAAACTAAAGAGGCTGTCGCTTTTCTGAGGAGGATCAAGGCTTTCGATGATGTTAAGAAGGTCTACAAAAGCCAAAGGATGAGGGCCGGTAAAGGTAAGATGCGTAACCGCAGACGCATCCAAAGGAAAGGACCCTTGATCGTCTACCACAAGGATGCCGGTCTCAAGAAGGCCTTCAGGAACATTCCCGGAGTCGACCTCATGAACGTCGAGAAACTGAACCTTTTGAAATTCGCCCCCGGTGGTCACGTAGGACGTTTCGTCATCTGGACCCAGTCCGCTTTCCAGAGACTGGACAAACTTTTCGGTACATACAAAGCAGCTTCGGGCGAAAAGAAGGGTTACACTCTTCCTCAGCCTAAGATGGCCAACACCGATTTGTCCAGGTTGTTGAAATCCGACGAGATCAAGAAGGTGCTCCGTGCTCCTCAGAAGAAGATCGTACGCAGTGTACGTCGTCTTAACCCACTTACCAACACCAAGGCTATGTTGAAATTGAACCCATATGCCGCAGTTCTCAAGCGTGAAGCCATCCTATCGGCACAAAAGAGGCAGTTGGCCAGAGAAGAAGCCCTTGCCAAAAAACGCGGGATCACCTTACCAGCTGATCATGCTGTAAACAGGACAGC |
| *RPS18* | AAAATGTCGCTGGTGATTCCAGATAAATTTCAACATATTCTCCGTATCCTCGGTACCAATATCGATGGAAAAAGGAACGTTATGTTCGCCCTCACCGCCATCAAAGGTGTAGGTCGTCGTTATGCCAATATTATCTTGAAAAAGGCTGATGTTGATTTACGCAAAAGGGCTGGAGAATGCTCCGACGAAGAAGTCGAGAAAATTATTACTATCATGTCTAACCCAAGGCAGTACAAAATCCCAGATTGGTTCTTGAATAGACAAAAGGATATTGTTGATGGTAAATATTCTCAGTTGACTTCATCAGCTTTAGACTCCAAATTGCGTGAAGATTTGGAACGTATGAAGAAAATCCGTGCCCACAGAGGCTTGCGTCACTACTGGGGTCTGAGAGTGCGTGGTCAACACACCAAAACAACCGGAAGACGTGGAAGAACTGTTGGTGTATCCAAGAAGAAGTAAATGTTTACTTTTATTTTATATGTAAGATAATAAAA |
| *HSP90* | ATCAAACATACGAGCGAGTTCGTGGTGAGCTGAGTCTCTCTTGAAAGTGTTACTTAAAGTGAATTTGTGCTTATTTTCTGAAATTAAATAAAATCCAACAAGATGCCTGAAGAAGTTCAGAATGGAGAAGTTGAAACCTTCGCCTTCCAAGCTGAAATTGCTCAGTTGATGAGCTTGATCATCAACACTTTCTACTCAAACAAAGAAATCTTCCTTAGGGAGTTGATCTCAAACTCTTCTGATGCTTTGGACAAGATCCGTTACCAATCCCTCACCAATCCATCATGTTTGGATTCTGGAAAGGACCTCTACATCAAAATCATTCCAAACAAAAACGATGGCACACTTACCATTATTGATACTGGTATTGGTATGACCAAGGCCGATTTAGTCAACAATTTGGGTACCATTGCCAAGTCCGGAACAAAGGCCTTCATGGAAGCTCTGCAAGCCGGTGCCGATATTAGCATGATTGGTCAATTTGGTGTAGGTTTCTACTCCGCCTATCTCGTTGCTGACAGAGTAACCGTTGTATCCAAAAACAACGATGATGAACAATACATCTGGGAATCATCCGCCGGTGGCAGCTTCACTGTTCGCACAGACCTTGGAGAACCACTTGGTAGAGGTACAAAGATCGTTCTTCACATCAAAGAAGATCAATCAGAATTCTTGGAAGAACACAAAATCAAAGAAATCGTAAAGAAACACTCCCAATTCATTGGATATCCCATCAAATTATTGGTAGAGAAAGAACGTGAAAAGGAATTGAGCGAAGATGAAGCCGAGGAAGAAAAGAAAGATGAAGAAGCAAGCGAAGAATCCGACAAACCCAAAATTGAAGATGTAGGTGAAGATGAGGAAGAAGATAAGGAAAAGAAGAAGAAAAAGAAGACCATCAAAGAAAAGTACACTGAAGATGAAGAACTCAACAAGACCAAACCTATTTGGACCAGAAACGCTGATGATATCTCTCAAGAAGAATATGGTGAATTCTACAAATCTTTGACCAACGATTGGGAAGATCATTTGGCCGTCAAGCACTTCAGCGTTGAAGGACAGTTGGAATTCAGAGCTCTGCTCTTCGTTCCACGTAGAGTACCATTCGATCTTTTTGAAAACAAGAAACGCAAGAACAACATCAAATTGTATGTACGCAGAGTTTTCATCATGGACAACTGCGAAGAATTGATTCCTGAATATTTGAACTTCATCAAGGGTGTTGTAGACTCTGAAGATTTACCCTTGAACATTTCCAGAGAAATGTTGCAACAGAACAAGATTCTTAAAGTCATCCGTAAGAATTTGGTCAAGAAATGCATGGAACTTTTCGATGAACTCACCGAAGACAAAGAGAACTTCAAGAAATTCTACGAACAGTTCTCAAAGAATCTCAAACTGGGTATCCACGAAGATTCTGCAAACAGATCAAAATTGGCTGAATTCCTCCGTTACCACACCTCTGCAAGCGGAGATGAAGCATGTTCTCTCAAAGACTACGTAAGCAGAATGAAGCAAAACCAGAAGAGCATTTACTACTTAACTGGAGAAAGCAAAGAACAAGTTGCCAACTCAGTATTCGTTGAACGTGTCAAGAAGCGTGGATTCGAAGTTGTCTACATGACCGAACCAATCGATGAATACGTTGTACAACAACTTAAGGAATACGACGGCAAAACATTGGTTTCTGTAACCAAGGAAGGTTTGGAATTGCCCGAAGATGAGGAAGAAAAGAAGAAGAGGGAAGAGGACAAAGCTAAATTCGAAGGTCTCTGCAAAGTTATCAAGAGCATTCTCGATAACAAAGTAGAAAAAGTTGTTGTTTCAAACAGACTGGTTGAATCTCCATGCTGTATTGTAACTTCCCAATATGGATGGACAGCCAACATGGAACGTATCATGAAAGCACAAGCTCTCCGAGACACATCCACAATGGGCTACATGTCCGCAAAGAAACATCTTGAAATTAATCCAGACCATCCAATTGTAGAGAACTTAAGACAAAAGGCTGAAGCCGACAAAAACGACAAGGCTGTTAAAGATTTAGTAATTCTTCTTTTTGAGACTGCACTTCTTAGCTCTGGATTCACTTTGGACGAACCACAAGTACATGCTTCTAGAATATACAGAATGATCAAATTAGGTTTGGGTATTGATGAAGAAGAATCCATGGTAGTCGAGGAACCTTCCGCTGATGCTCCTGCAGCTGAAGCCGGAGATTCAGAAGATGCGTCAAGAATGGAAGAAGTCGATTAAGCTACTCTATACTTTTAATATGTGTTATACTAATGAACATGTGTTTTAGTATGTTCCATCATTCCTAATAAGTTATTTTTTTATAGTACCTATCTCGAAAGAATAATTTTTGGCTGTGTTCCAACAAAGATTTCTGCTCGTACTGATTATTGTTAAAGTATTTGAGAATAAAATGTCATCATATTTA |
| *ArgK* | CGACTAAGTATAGGGCGATTGGGCCCTCTAGATGCATGCTCGAGCGGCCGCCAGTGTGATGGATATCTGCAGAATTGCCCTTCCTGTTCGACCCTATCATCGAGGACTATCATGGTGGATTCAAAAAGACTGACAAACATCCCCCAAGGGACTTCGGGGATGTTAACGTATTTGGTAACTTGGATCCTGCCGGGGAATATATTGTTTCAACTCGTGTAAGATGCGGACGTTCTTTGGAGGGATACCCCTTCAACCCTTGCTTAACTGAAGAGCAATACAAGGAGATGGAACAGAAGGTTTCGTCCACTTTATCTGGACTCGACGATGAACTCAAAGGTACTTTCTACCCATTGACCGGTATGGACAAGGAAACCCAGCAGAAACTTATTGACGATCACTTCTTGTTCAAGGAAGGTGACAGATTCTTACAGGCAGCAAACGCTTGCAGATTCTGGCCTTCAGGTCGTGGTATATTCCATAACGACGCCAAGACCTTCTTGGTCTGGTGCAACGAAGAAGACCATCTCAGAATCATCTCCATGCAGATGGGTGGTGATCTTGGCCAAGTTTACCGCCGACTTGTCTCTGCTGTTAACGAAATCGAAAAGCGCCTACCATTCTCACACAACGACAGACTTGGCTTCCTCACATTCTGCCCAACTAACTTGGGCACCACTGTCAGAGCTTCTGTCCACATCAAGGTACCGAAATTGGCCTCCAACAAGGCCAAACTTGACGAGATTGCCGGCAAATTCAATCTGCAAGTCCGTGGTACTCGTGGCGAGCACACCGAAGCTGAAGGTGGTATCTACGACAAGGGCAATTCCAGCACACTGGCCGGCCGTTACTAGTGGATCCGAGCTCGGTACCAAGCTTGGCGTAATCATGGTCATAGCTGGTTCCTGGGTGAAATTGGTATCCGCTCACAATTCCACACAACTTACGAACCCGGAAGCATAAAGTGTAAAGCCTGGGGTGCCTAATGAATGAGCTAACTCCAA |
| *V-ATPase A* | GACTCCTCAGAACCTTGTGACAAATTTACTCTTCAGGTTTACGTCGGAAGCTCATATTTTCAGTCTTACGGGAAAATTGGGTGAGGTTTTGGTGATCTTGTGGTGTTTTTCTGTTTTGTGACCACCAGCAAAGTTCAAAGTACTTCAAAATGTCGAATTTACCCAAAATACGGGACGAAGACCGAGAGTCTAACTATGGTTATGTCCATGCAGTTTCAGGACCTGTCGTAACTGCTGAAAAAATGTGTGGGTCTGCTATGTACGAGCTCGTTCGAGTTGGTTACTTCGAACTGGTGGGTGAAATTATTCGTCTTGAAGGAGATATGGCCACAATTCAGGTATACGAGGAAACTTCTGGCGTAACCGTTGGTGATCCTGTACTGCGTACAGGAAAACCATTGTCTGTTGAATTGGGTCCTGGTATTATGGGTTCTATTTTTGATGGTATCCAGCGTCCATTGAGAGACATCAATGTCCTCACAGAAAGTATCTACATTCCTAAGGGTATTAACGTACCTTGTCTATCTAGGACTGCAAAATGGGACTTCAATCCTTCTAATATCAAATTGGGATCACATTTAACTGGAGGCGATATCTATGGTCTCGTCCATGAAAACACCTTGGTCAAACAAAAATTGATGCTGCCTCCAAAATCTAAAGGTACAGTTACATATATAGCAGAACCAGGAAGCTACACTGTTGATGACGTCGTCCTGGAAACCGAATTCGATGGCGAGCGCACCAAATACACCATGTTGCAAGTCTGGCCTGTCCGTCAGCCACGTCCAGTCAGTGAAAAACTGCCTGCCAATCATCCACTGCTTACCGGACAAAGAGTTTTGGACTCTCTGTTCCCATGTGTACAGGGTGGTACCACTGCTATCCCTGGTGCTTTCGGTTGTGGAAAAACTGTCATCTCTCAATCCTTGTCCAAATACTCCAACTCCGACGTCATTATCTACGTAGGTTGCGGAGAGAGAGGTAACGAAATGTCTGAAGTACTTCGGGACTTCCCCGAACTGACAGTCGAGATCGATGGTCAGACCGAATCAATCATGAAACGTACCGCTCTGGTAGCCAACACCTCCAACATGCCTGTCGCCGCTCGTGAAGCATCTATTTACACCGGTATCACTTTGTCAGAATACTTCAGAGACATGGGTTACAACGTGTCCATGATGGCCGATTCCACCTCTCGTTGGGCCGAGGCTCTGAGAGAAATCTCCGGTCGTCTGGCTGAAATGCCTGCCGATTCTGGTTACCCAGCCTACTTGGGGGCCCGTCTCGCCTCCTTCTACGAACGTGCAGGAAGAGTCAAATGTCTGGGTAATCCAGATCGTGAGGGTTCCGTTTCCATCGTAGGAGCCGTGTCGCCACCTGGTGGTGACTTCTCAGATCCTGTCACATCTGCCACTCTTGGTATCGTACAGGTGTTCTGGGGTTTGGACAAAAAGTTGGCCCAACGTAAACATTTCCCCTCCATCAACTGGCTGATTTCTTACTCGAAGTACACGAGAGCTTTGGACGATTTCTACGATAAGAACTTTGCTGAGTTTGTGCCGTTGAGAACTAAGGTCAAGGAAATCTTGCAGGAAGAAGAAGACTTGTCCGAAATTGTGCAATTGGTAGGAAAGGCATCACTTGCTGAAACTGACAAAATCACCCTGGAAGTAGCCAAACTTCTGAAAGAAGATTTCCTTCAACAAAACTCATACTCATCCTACGATAGGTTCTGCCCCTTCTATAAGACAGTGGGTATGTTGAAAAACATGATTGGTCTTTATGACATGGCAAGGCACGCAGTTGAAACAACCGCTCAATCTGAAAACAAAATCACATGGAATGTAATCAGGGAAGCAATGAGCAATATTTTGTACCAACTCAGCAGTATGAAATTCAAAGATCCTGTTAAAGATGGGGAAGCGAAAATTAAAGCTGATTTCGATCAACTTTATGAAGATATACAGCAAGCTTTCAGAAACTTAGAAGATTAGGTACAATAATATATGTTTTAGAAATCAGAAAGGCCTGCGGAACTGTTTGATCTAATTTTTTTGGTGAACCATATCCAAATTCGTATGAATTGGCCATAATTATATACTGATAATAATTCATTGTAAGTGTACAATACCATGTATATATATAAGGTGGATCAAGATTATATCTGAAACTTACTGAATGGTATTTTCACCAATGAGACTGCTGCACAAATTTTCAAGAAATAACTGTACTCTCAACAGTTAACAGAAAGTCCCA |
